# Deep learning-based fully automated Z-axis coverage range definition from scout scans to eliminate overscanning in chest CT imaging

**DOI:** 10.1186/s13244-021-01105-3

**Published:** 2021-11-06

**Authors:** Yazdan Salimi, Isaac Shiri, Azadeh Akhavanallaf, Zahra Mansouri, Abdollah Saberi Manesh, Amirhossein Sanaat, Masoumeh Pakbin, Dariush Askari, Saleh Sandoughdaran, Ehsan Sharifipour, Hossein Arabi, Habib Zaidi

**Affiliations:** 1 Division of Nuclear Medicine and Molecular Imaging, Geneva University Hospital, 1211 Geneva, Switzerland; 2 Department of Biomedical Engineering and Medical Physics, Shahid Beheshti University of Medical Sciences, Tehran, Iran; 3 Imaging Department, Qom University of Medical Sciences, Qom, Iran; 4 Department of Radiology Technology, Shahid Beheshti University of Medical, Tehran, Iran; 5 Men’s Health and Reproductive Health Research Center, Shahid Beheshti University of Medical Sciences, Tehran, Iran; 6 Neuroscience Research Center, Qom University of Medical Sciences, Qom, Iran; 7 Geneva University Neurocenter, Geneva University, Geneva, Switzerland; 8 Department of Nuclear Medicine and Molecular Imaging, University of Groningen, University Medical Center Groningen, Groningen, Netherlands; 9 Department of Nuclear Medicine, University of Southern Denmark, Odense, Denmark

**Keywords:** CT, Radiation dose, Overscanning, Deep learning, Chest imaging

## Abstract

**Background:**

Despite the prevalence of chest CT in the clinic, concerns about unoptimized protocols delivering high radiation doses to patients still remain. This study aimed to assess the additional radiation dose associated with overscanning in chest CT and to develop an automated deep learning-assisted scan range selection technique to reduce radiation dose to patients.

**Results:**

A significant overscanning range (31 ± 24) mm was observed in clinical setting for over 95% of the cases. The average Dice coefficient for lung segmentation was 0.96 and 0.97 for anterior–posterior (AP) and lateral projections, respectively. By considering the exact lung coverage as the ground truth, and AP and lateral projections as input, The DL-based approach resulted in errors of 0.08 ± 1.46 and − 1.5 ± 4.1 mm in superior and inferior directions, respectively. In contrast, the error on external scout views was − 0.7 ± 4.08 and 0.01 ± 14.97 mm for superior and inferior directions, respectively.The ED reduction achieved by automated scan range selection was 21% in the test group. The evaluation of a large multi-centric chest CT dataset revealed unnecessary ED of more than 2 mSv per scan and 67% increase in the thyroid absorbed dose.

**Conclusion:**

The proposed DL-based solution outperformed previous automatic methods with acceptable accuracy, even in complicated and challenging cases. The generizability of the model was demonstrated by fine-tuning the model on AP scout views and achieving acceptable results. The method can reduce the unoptimized dose to patients by exclunding unnecessary organs from field of view.

**Supplementary Information:**

The online version contains supplementary material available at 10.1186/s13244-021-01105-3.

## Key points

- Overscanning is a common problem (more than 95% of the cases) occurring mostly in the inferior direction in clinical practice, leading to additional unnecessary radiation dose in chest CT.
- We developed an accurate and robust automated method for scan range delimitation trained on a large dataset with acceptable reproducibility.
- Our proposed deep learning-guided algorithm could potentially reduce patient's radiation dose by up to 21%.

## Introduction

Computed Tomography (CT) has been during recent decades and remains presently one of the most prevalent technologies used in diagnostic imaging. As the utilization of CT scanning is increasing, this modality currently accounts for the primary source of medical radiation exposure to the population. In this regard, recent innovations have focused on optimizing radiation dose associated with this technology, e.g., the new generation of detectors [1], adaptive collimation, iterative image reconstruction, adaptive voltage, and exposure control, have strongly reduced CT radiation dose. Yet, "*CT is still not a low-dose imaging modality*" [2]. Although several functionalities, such as automatic tube voltage selection and tube current modulation (TCM), have been successfully implemented on CT scanners to optimize radiation exposure, some scanning parameters impacting image quality and radiation dose, such as patient positioning, scan range choice, and localizer scan parameters are manually selected by technologists [3, 4]. In this light, developing and establishing automated scanning procedures to minimize human error and homogenize imaging protocols is desirable [5, 6].

Hitherto, there is no commercialy available systems enabling automatic scan range selection. In current clinical practice, the scan length is commonly selected based on task-specific landmarks extracted from two-dimensional (2D) anterior–posterior (AP) or lateral scout scans. This manual procedure is prone to human error depending on the technologist's experience and the medical center's workload. Zanca et al*.* reported that up to 80% of thoraco-abdominal CT examinations suffered from overscanning with an average of 1.8 and 2.9 cm extra-scanning length at the superior and inferior directions, respectively [7]. Schwartz et al*.* reported a maximum of 60% incidence of overscanning in chest CT with substantial variability among the different institutions causes up to 50% dose increasing in specific organs such as the thyroid [8]. Yar et al*.* reported that more than 60% of CT examinations had more than the necessary coverage, especially in the inferior direction [6]. Conversely, Cohen et al*.* reported a high frequency (95%) of overscanning in chest CT and demonstrated a strong correlation with the workload of technologists [9, 10].

Recent advances in artificial intelligence, specifically deep learning (DL), have revolutionized the domain of computer vision and image processing. In the context of medical imaging, DL has been successfully deployed in challenging tasks, such as image segmentation/interpretation, cross-modality image translation, image denoising, radiotherapy treatment planning, and outcome prediction [11–13].

Zhang et al*.* developed a machine learning technique to detect landmarks on the localizer image to indicate the desired spiral scan limits resulting in errors around 6 mm [14]. Recently, Colevray et al*.* developed a convolutional neural network (CNN) model for the assessment of overscanning length associated with lung CT scanning, which showed a good agreement with radiologists' evaluation (kappa = 0.98) [15]. More recently, Demircioglu et al*.* developed a conditional generative adversarial network (cGAN) for the delimitation of CT scan range by training the model by radiologist selected scan ranges and yielded the average error of 1.8 and 3.3 mm in superior and inferior directions [16].

In this work, we developed an automated workflow for task-specific scan range selection and retrospective evaluation of overscanning on a large cohort of a multi-centric and multi-purpose clinical database of thoracic CT examinations. The impact of overscanning on patients' effective dose was investigated through personalized dosimetry of the considered cohort.

## Materials and methods

### Method description

**Fig. 1:**
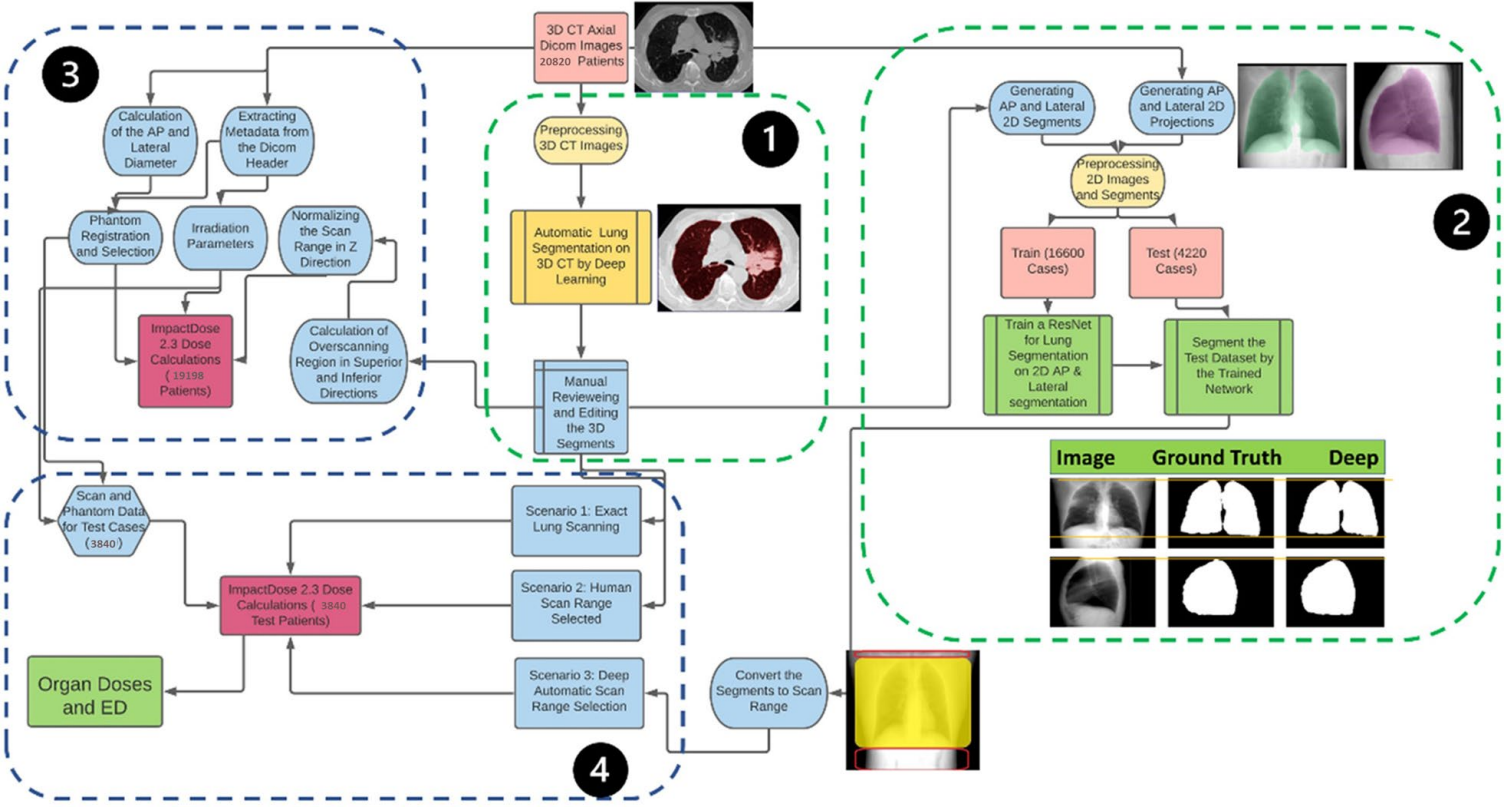
The workflow adopted in this study protocol. 1) 3D lung segmentation from CT images, 2) generation of 2D projections and 2D lung segments from 3D images and training the deep learning network for semantic segmentation, 3) extracting the metadata and estimating the dosimetric impact of overscanning for all 20,820 patients. 4) Calculation of the ED and organ doses based on three scenarios described in the text

A fully automated DL-based algorithm was used to segment the 3D CT images [17]. Based on the acquired 3D lung masks, the exact scan range was determined such that the axial slices containing the lung masks with a margin of one voxel in superior (cranial) and inferior (caudal) directions were included. In the second step, 2D projections of CT images in AP and lateral views were generated, representing properties of scout scans. Likewise, 2D lung masks were generated from 3D lung segmentations (reference segmentation) to train a DL model for the prediction of 2D lung masks from 2D localizer images. The 2D AP and lateral images segmented using the trained network were converted to the scan range by considering the first pixel in the craniocaudal direction as the upper limit (superior) and the last pixel containing the lung segment as the lower limit (inferior) for the scan. The scan ranges selected based on AP and lateral images are referred to as DL_AP and DL_Lat, respectively. Hence, overscanning in superior and inferior directions was calculated and compared with the reference manually selected ranges during imaging in a clinical situation (via human intervention). The differences between the ground truth limits and the DL-predicted limits were considered as the error. In contrast, errors less than zero are related to the exclusion of some lung slices, whereas positive error values indicate overscanning. The radiation dose delivered to organs and total body effective dose (ED) was then calculated for different scenarios. We have performed fine-tuning on our trained network using 3423 scout views to evaluate the reproducibility of our model on real localizer datasets. In the following sections, we explain these steps in more detail. Figure 1 depicts the detailed process in a multi-step workflow.

### Study population

**Fig. 2:**
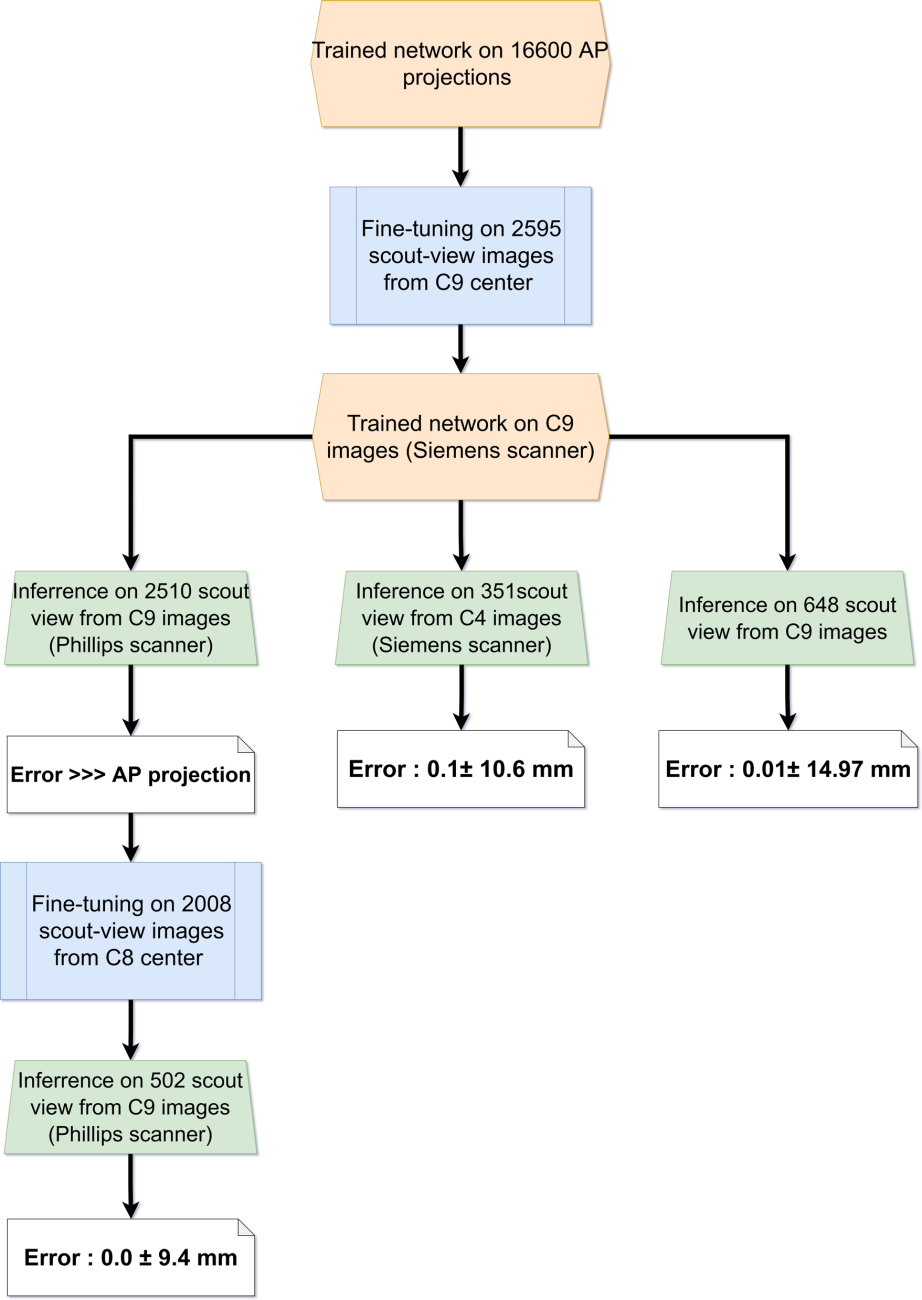
Flowchart for analysis of scout view images included in this study after fine tuning on C9 images and testing the network on two other datasets of C4 and C8

This work included a large-scale retrospective cohort of 20,820 chest CT images. The major part was from 9 centers acquired on ten different CT scanner models in Iran (C1 to C9, 12,146 cases). The patients were referred for assessment, follow-up, and roll-out of COVID-19. Besides, three online databases of TCIA chest CT (D1, 600 cases) [18, 19], Russia chest CT for assessment of COVID-19 (D2, 1022 cases) [20], and RSNA chest CT images (D3, 7052 cases) [21] were included. D1 and D3 databases were created from multiple pathologies. Table 1 summarizes the demographic information of the study population and acquisition parameters. Besides, the scout views were collected for dataset C9 to assess the generalizability of our model on scout views in clinical setting. According to the flowchart (Fig. 2), we initially trained a network on a large dataset consisting of over 16,600 cases for the prediction of scan range from 2D projection images (generated from 3D axial slices). Thereafter, the developed model, was fine-tuned on a real scout scan datasets obtained from a single scanner (C8, Siemens Emotion). Since pixel intensities of scout images are vendor-specific, we tested this model on two different scanners, namely the Siemens Somatom and Philips from different centers (C4 and C8, respectively). In the case of the Siemens scanner, i.e. same vendor but different model (C4, 351 cases), the results were consistent with the primary reported results based on C9 (Table 1). However, for datasets obtained from the Philips scanner, the model trained on the Siemens scanner predicted the scan range with overall larger error than our initial reports on C9. In this regard, we further fine-tuned our model for the Philips scanner by dividing the database obtained from C8 into three groups of train, validation, and test sets. The results summarized in Table 1 were comparable with those reported from C9 and C4.

**Table 1:** Patients’ demographics and CT scanner manufacturer and acquisition parameters plus overscanning ranges selected by the technologist in both directions in the whole dataset categorized by centers

Database	Gender		*D*_*AP*_ (cm)	*D*_*Lat*_ (cm)	Scanner Manufacturer	Tube current (mA)	CTDI_vol_ (mGy)	SSDE (mGy)	DLP (mGy.cm)	Overscanning (mm)	
										Superior	Inferior
	Male	Female	Mean ± STD	Mean ± STD		Mean ± STD	Mean ± STD	Mean ± STD	Mean ± STD	Mean ± STD	Mean ± STD
C1	260	223	23.3 ± 2.3	31.1 ± 3.6	GE	186.2 ± 34.0	8.47 ± 0.53	11.69 ± 0.85	97 ± 24	19.6 ± 10.9	42.3 ± 29.9
C2	759	564	24.1 ± 2.7	32.0 ± 3.7	GE	228.4 ± 27.2	6.82 ± 1.02	9.04 ± 0.98	19 ± 15	2.27 ± 1.71	1.89 ± 2.39
C3	435	303	23.7 ± 2.7	31.2 ± 3.6	Siemens	123.6 ± 9.6	3.40 ± 0.42	4.65 ± 0.6	33 ± 11	25.0 ± 19.0	54.0 ± 37.2
C4	978	929	23.2 ± 3.0	31.7 ± 4.0	Siemens	147.1 ± 43.2	6.13 ± 2.07	8.21 ± 2.14	103 ± 36	28.0 ± 11.5	33.7 ± 21.3
C5	196	171	22.7 ± 3.1	31.4 ± 4.1	Siemens	151.1 ± 47.8	6.22 ± 2.17	8.48 ± 2.52	106 ± 39	29.2 ± 12.3	37.8 ± 22.6
C6	465	412	24.2 ± 3.0	33.1 ± 3.7	Siemens	166.2 ± 51.3	4.30 ± 1.54	5.49 ± 1.50	79 ± 29	30.0 ± 11.4	39.6 ± 22.1
C7	869	779	22.7 ± 2.8	30.4 ± 3.9	NMS	220.7 ± 27.9	8.92 ± 1.40	12.61 ± 1.89	199 ± 33	30.9 ± 12.2	35.3 ± 26.1
C8	518	530	23.0 ± 3.8	31.5 ± 5.0	Phillips	89.3 ± 48.2	3.73 ± 2.09	5.05 ± 2.62	59 ± 34	23.5 ± 13.2	24.8 ± 21.7
C9	1489	1300	23.3 ± 2.9	31.2 ± 3.8	Siemens	174.0 ± 37.5	5.91 ± 1.52	7.99 ± 1.49	149 ± 42	25.7 ± 12.3	37.3 ± 21.9
C10	594	372	23.2 ± 3.2	31.0 ± 4.6	Siemens	210.7 ± 39.6	5.57 ± 1.21	7.69 ± 1.65	47 ± 27	24.0 ± 17.9	37.9 ± 39.1
D1	NA	NA	20.4 ± 3.1	26.7 ± 5.4	NA	NA	NA	NA	NA	32.6 ± 10.9	26.0 ± 17.2
D2	NA	NA	23.3 ± 3.3	29.7 ± 4.8	NA	NA	NA	NA	NA	34.7 ± 13.3	34.8 ± 22.4
D3	NA	NA	23.0 ± 3.2	30.6 ± 4.2	NA	424.0 ± 191.8	NA	NA	NA	22.1 ± 12.4	40.6 ± 35.6

Considerable variability in scanner model, pathologic conditions, patient positioning and CT imaging protocols among the centers was observed, which caused significant variability in image quality and patient radiation dose. Volumetric CT Dose Index (CTDI_vol_) as a parameter representing a vendor-free metric of radiation exposure and consequently image quality was reported to reflect the variability of our dataset.

### 3D lung segmentation

A deep residual network architecture previously developed and validated by our group was deployed for lung segmentation from 3D axial CT images [17]. The whole lung from the apex to costophrenic sinuses was included. The segmented lung masks were visually reviewed to avoid noticeable errors.

### 2D projection generation and preprocessing

Since localizer images were available only for the C9 dataset, we projected the 3D CT images by summing the slices in lateral and AP directions to generate a 2D image mimicking the localizer image as reported earlier in a number of studies [22, 23]. Accordingly, the lung masks on 3D images were projected on the same AP and lateral views as the CT images. The 2D masks were fed into the network as the ground truth for 2D semantic segmentation (Fig. 1). The intensity of 2D images was normalized, and the whole dataset was resized to 256 × 256 matrix size for efficient segmentation.

### Network architecture

We implemented a 2D deep residual neural network for semantic segmentation of 2D projections in PyTorch [24]. A modified 2D version of a previous deep residual neural network including 20 convolutional layers was implemented [25]. Different feature levels were extracted by the dilation factor concept, where each two layers were connected using a residual connection. The data were split into train (80%) and external validation sets (20%) for each center and scanner to include images from all the databases in the training and validation group. The external validation group remained untouched during the training. The body fine-tuning approach was employed to transfer the weights from the network trained on 16′600 AP projections to a new network to be fine-tuned by the real scout views.

### Scout-view preparation and training

The AP localizer images were collected for patients in center C9, and the same procedure for lung segmentation was implemented on axial slices. The 3D lung segments were transferred to the scout-view (localizer) by implementing image distortion due to magnification on 3D lung segments. The total of 3243 AP localizers were split into 70% (2270 cases) train, 10% (325 cases) validation, and 20% (648 cases) test sets. The error in the determination of superior and inferior lung boundaries was evaluated as performed on the projections.

### Personalized dosimetry

To estimate personalized organ absorbed dose from chest CT scans, we adopted a habitus-specific organ-level dosimetry approach. This approach deployed sets of pre-calculated organ-level dose tables based on a computational phantom library, by considering the scan parameters, adoption of the phantom to the patient size and CTDI_vol_ the organ doses are close to a personalized parameter [26]. In this regard, patient-specific anatomical metrics along with acquisition parameters were extracted from DICOM CT images. Age, sex, and effective diameter ($D_{eff} = \sqrt {D_{Lat} \times D_{AP} }$) representing individualized anatomical features, tube voltage, effective tube current–time product (mAs/pitch factor), scanner model, and CTDI_vol_ were fed to the ImPactDose software (CT Imaging GmbH, Germany) to estimate patient organ absorbed doses associated with CT examination [27]. To address the dosimetric impact of vendor-specific x-ray beam quality, normalized CTDI_vol_ (CTDI_vol_/100 mAs) was introduced into ImPactDose. Since organ dose variations pertinent to scanner-specific simulation parameters are approximately the same as variations in scanner-specific CTDI_vol_ [28–30], we considered the CTDI_vol_ as scanner specific parameter in our calculations. DICOM images of the online databases were anonymized and essential information for dose calculation was missing. As shown in Table 2, the tube current was available for the RSNA (D3) database. Hence, we excluded databases of D1 and D2 from dose calculations. As such, dose calculation was performed for 19,198 patients to assess the additional dose due to overscanning. Besides, the radiation dose was calculated for 3840 patients consisting the external validation group. Because of missing gender information for the D3 dataset, in estimating the dose for D3 cases, organ and effective doses were calculated considering both genders, and the average value reported.

**Table 2:** The additional effective dose delivered to patients due to overscanning in superior and inferior directions in the different centers (mSv)

	Additional ED superior (mSv)						Additional ED inferior (mSv)				
Database	Mean ± STD	min	Q1	Q3	Max	Mean	STD	Min	Q1	Q3	Max
C1	0.92 ± 0.28	0.18	0.88	1.10	1.91	2.08	0.55	0.51	1.90	2.43	3.41
C2	0.17 ± 0.17	0.02	0.04	0.24	0.80	0.33	0.34	0.04	0.10	0.47	3.71
C3	0.28 ± 0.07	0.15	0.22	0.34	0.57	0.77	0.24	0.39	0.58	0.91	1.65
C4	1.17 ± 0.40	0.07	0.90	1.35	3.35	2.13	0.59	0.19	1.74	2.43	5.34
C5	1.21 ± 0.44	0.31	0.92	1.40	3.31	2.24	0.69	0.84	1.77	2.53	5.16
C6	0.88 ± 0.29	0.10	0.67	1.03	1.96	1.55	0.44	0.37	1.24	1.80	3.12
C7	2.62 ± 0.71	0.60	1.99	3.09	5.51	4.29	0.83	1.33	3.65	4.81	7.94
C8	0.66 ± 0.36	0.05	0.42	0.78	2.68	1.24	0.68	0.13	0.78	1.46	5.41
C9	1.93 ± 0.65	0.10	1.45	2.51	3.92	2.87	0.71	0.00	2.39	3.43	5.18
C10	0.42 ± 0.37	0.07	0.21	0.51	2.93	1.00	0.60	0.22	0.61	1.18	5.61
D3	0.65 ± 0.56	0.10	0.39	0.77	8.05	2.33	1.25	0.46	1.56	2.77	14.80

### Scan range selection

For dose calculation, three scan range selection scenarios were considered:

(i) Assuming exact scan range selection for total lung coverage without losing anything and without any overscanning. The scan range was selected according to the lung mask obtained from 3D images.
(ii) The actual scan range selected by the technologist during the CT examination.
(iii) Selecting the scan range based on 2D lung mask obtained from the neural network model on 2D segmentation after post-processing. The post-processing was performed only to remove segmented regions with less than a certain number of pixels while keeping the largest segment.

The lung position in the Z-direction for the ORNL phantom was extracted from the ImPactDose software user manual. The scan range in the superior and inferior parts of the lung recorded for the second and third scenarios was normalized according to the lung length of the patient and the phantom. TCM was considered for more accurate dose calculation. Hence, the tube current recorded in the DICOM header for each slice was averaged for the lung, superior, and inferior scan regions [31]. Size-specific dose estimate (SSDE) and effective diameter were calculated using the conversion factors reported in the AAPM report 220 [32]. The dose length product (DLP) was defined as the product of CTDI_vol_ and the scan range in each scenario. Radiation dose calculations were performed for 3840 patients constituting the external validation group (D1 and D2 were excluded) according to the three scenarios considering the ICRP 103 weighting factors [33]. Organ doses and ED were reported. For the above mentioned databases (D1 and D2), only the scan length was indicated.

The organ doses and ED due to overscanning in the superior and inferior directions selected by the operator for all 19,198 patients were calculated. The additional radiation dose due to scanning regions other than the lungs in both directions was estimated for all patients. The data were entered to ImPactDose in comma-separated value (CSV) format and the output saved for all patients. The ORNL phantom available in the software was modified according to the patient's lateral and AP diameters.

### Statistical analysis

We used SPSS software for statistical analysis. We employed the Kolmogorov–Smirnov test for the assessment of normal distribution. For evaluation of the differences between genders, direction, and scenarios, the Mann–Whitney test was employed. Spearman test was used to analyse the correlation between parameters and *p*-value < 0.05 was considered as threshold for statistically significant difference.

## Results

Table 1 summarizes the demographic information of the studied population consisting of gender, patients' AP and Lat dimeters, CT acquisition parameters, and individualized dose indices. A statistically significant correlation between the patient diameter and overscanning was observed. Overscanning occurred in 99% and 95% of the cases in the superior and inferior directions, respectively. Moreover, the extent of overscanning in the inferior direction was significantly larger (*p* < 0.001). Table 2 summarizes the additional radiation dose delivered to patients due to overscanning in clinical setting for the whole dataset (19,198 cases). As shown in Tables 1 and 2, there is noticeable variability among centers, either in terms of overscanning range or ED, while the additional radiation dose from inferior overscanning is more than superior overscanning. The magnitude of actual overscanning was larger in female patients (*p* < 0.001). In addition, there was a positive correlation between overscanning and patients' age (*p* < 0.05).

**Fig. 3:**
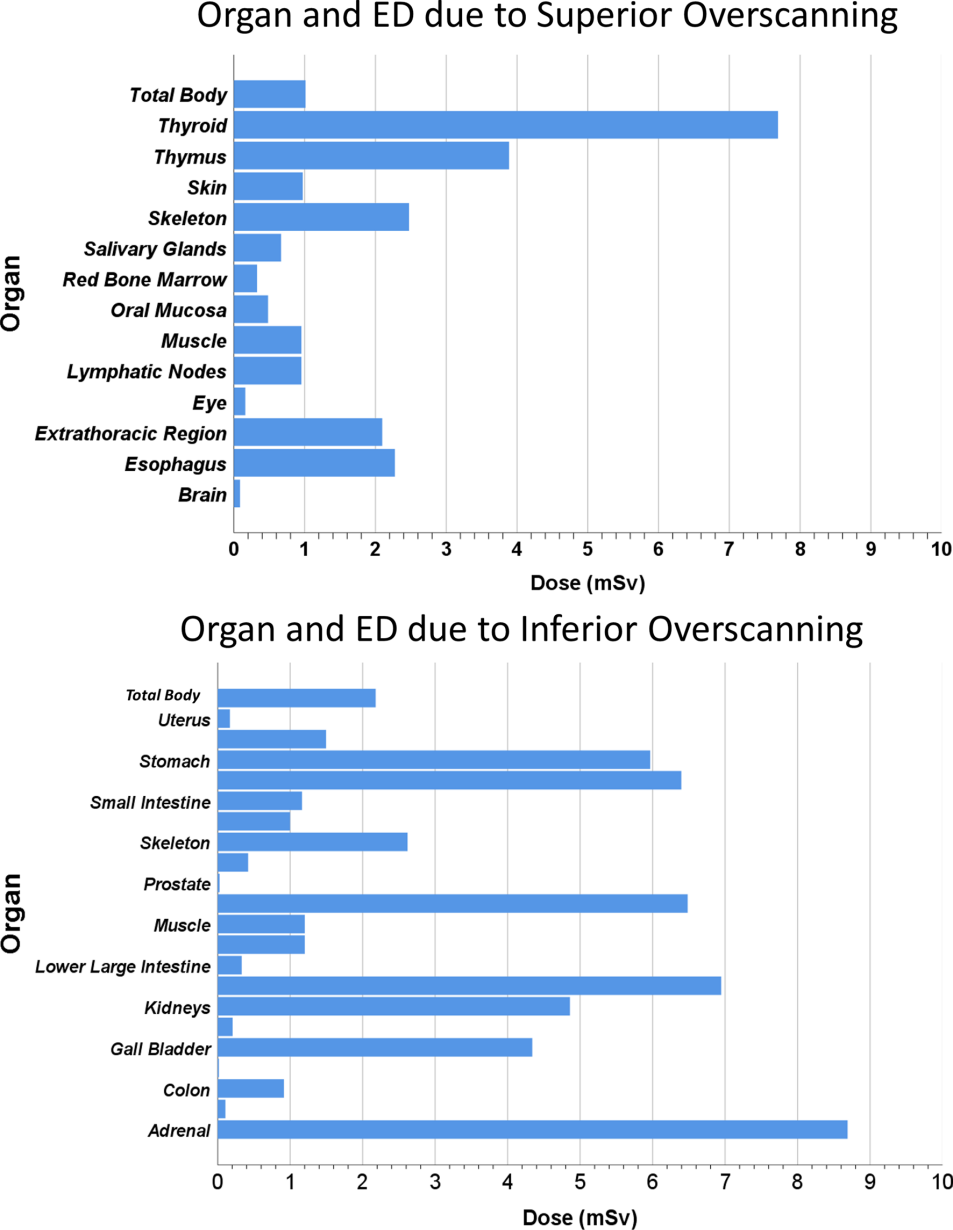
Additional radiation dose in terms for selected organs (in mGy) and effective dose (in mSv) due to overscanning in superior (left) and inferior (right)

Figure 3 shows the additional radiation dose burden in terms of total-body effective dose to patients due to overscanning in both directions.

### Deep learning network performance evaluation

**Fig. 4:**
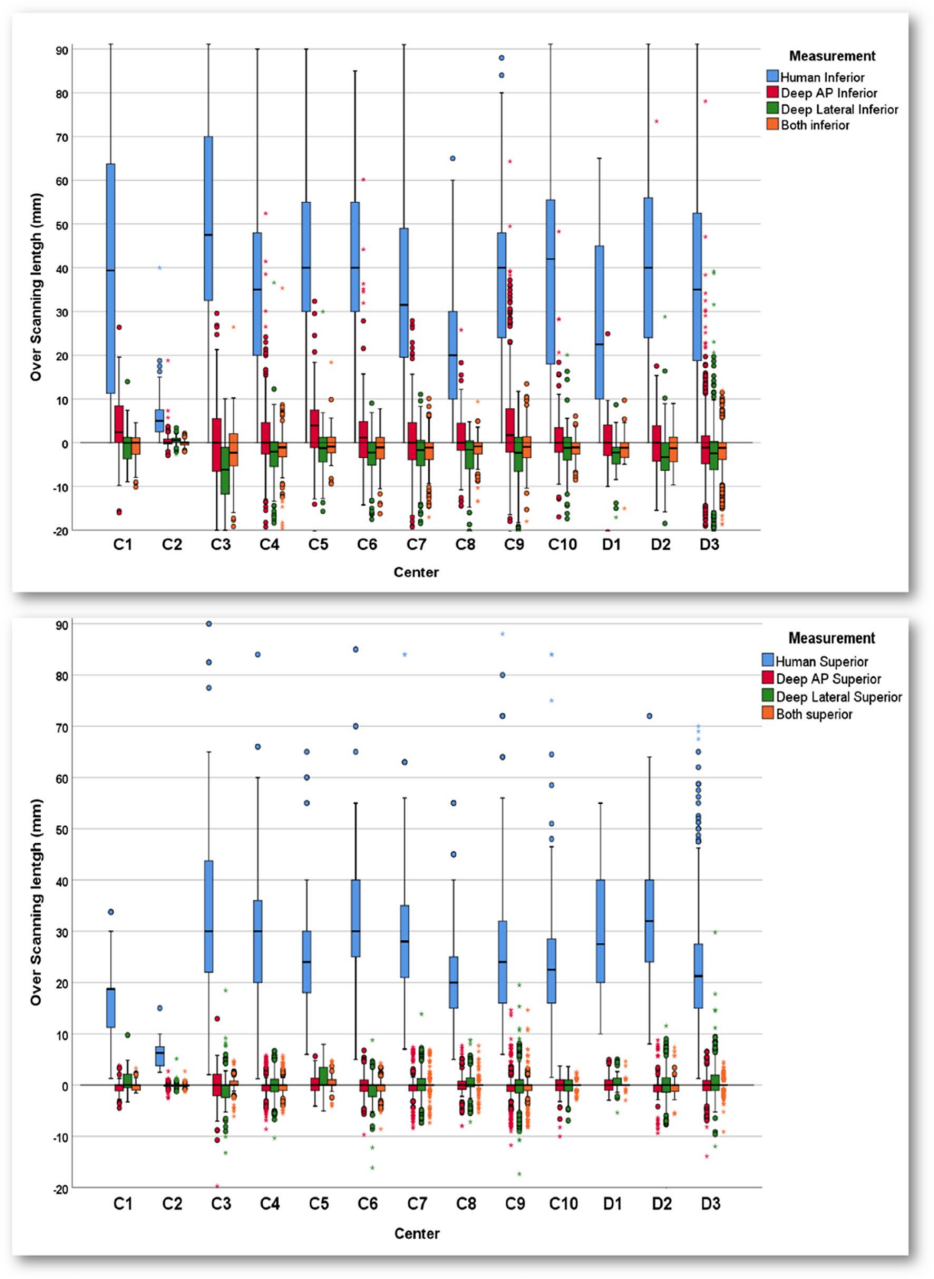
Error from the desired exact lung coverage in the superior (bottom) and inferior (top) directions according to the technologist (Human) performance and DL approach based on AP, lateral, and both projections for different categories of datasets

The average Dice factor for the external validation set consisting of 4220 patients (projections) was 0.96 ± 0.016 (Q1 = 0.95, Q3 = 0.981, max = 0.993) and 0.97 ± 0.02 (Q1 = 0.97, Q3 = 0.985, max 0.995) for AP and lateral projections, respectively. In addition, the Dice factor for 648 localizer images was 0.92 ± 0.03(Q1 = 0.90, Q3 = 0.95, max 0.97). Figure 4 presents the scan range selected by the operator (Human) and ranges selected based on AP, lateral, and both projections, where the zero-reference line is related to exact lung coverage without missing anything. The error averaged over all 4220 external cases was 0.08 ± 1.46 and − 1.5 ± 4.1 mm in superior and inferior directions, respectively. As presented in Table 3, the error was significantly larger in the inferior direction. The lateral projection-based scan range selection led to less absolute error in the inferior direction, which was not statistically significant (*p* = 0.32). However, for superior range selection, the absolute error was less based on the AP projection (*p* < 0.001). The details of scan ranges are presented in Table 2. The DL scan range error is significantly lower than human performance (*p* < 0.001). The DL method performance was excellent in superior delimitation for all centers and significantly better than the human selected range in the inferior direction, e.g., in C3 and D2 databases, the DL-based error is almost zero while the errors are 55 and 40 mm for human selection. The error for scout-view scan range selection was significantly less than human selection in the same C9 center and was comparable with external validation results for AP projections generated by averaging.

**Table 3:** The error and difference between the lung segment and the scan range selected by the technologist (Human) and DL network

Center	Superior error (mm)				Inferior error (mm)			
	Human	Deep AP	Deep Lateral	Deep Both	Human	Deep AP	Deep Lateral	Beep Both
C1	16.9 ± 7.9	− 0.3 ± 1.7	0.7 ± 2.2	− 0.1 ± 1.0	41.7 ± 35.5	2.3 ± 9.5	− 2.46 ± 9.9	− 1.5 ± 5.5
C2	6.3 ± 2.2	− 0.1 ± 0.5	− 0.1 ± 0.6	− 0.1 ± 0.3	5.3 ± 4.5	0.4 ± 1.6	− 0.54 ± 0.8	− 0.1 ± 0.6
C3	33.3 ± 19.7	− 0.3 ± 3.4	− 0.6 ± 3.8	0.1 ± 1.9	55.2 ± 32.7	0.0 ± 10.7	− 7.36 ± 10.2	− 2.4 ± 6.8
C4	29.2 ± 11.6	**0.0 ± 2.2**	− 0.2 ± 2.6	− 0.1 ± 1.5	34.2 ± 22.3	**1.0 ± 8.5**	− 3.06 ± 6.8	− 1.5 ± 4.8
C5	26.2 ± 12.6	0.4 ± 2.2	1.0 ± 3.0	0.3 ± 1.8	43.7 ± 22.6	3.8 ± 9.3	− 1.96 ± 7.0	− 0.2 ± 4.3
C6	33.1 ± 12.9	− 0.3 ± 2.6	− 0.8 ± 2.9	− 0.5 ± 1.7	41.3 ± 21.1	2.3 ± 10.7	− 3.06 ± 5.5	− 1.3 ± 3.9
C7	29.6 ± 10.3	− 0.1 ± 2.7	0.4 ± 2.9	0.1 ± 1.9	34.8 ± 23.6	0.3 ± 7.5	− 2.66 ± 5.4	− 1.8 ± 4.1
C8	21.7 ± 10.2	**0.1 ± 2.5**	0.5 ± 2.8	0.1 ± 1.7	22.1 ± 16.0	**0.9 ± 5.7**	− 3.76 ± 7.1	− 1.3 ± 3.0
C9	26.0 ± 9.2	− **0.4 ± 3.1**	− 0.7 ± 3.7	− 0.3 ± 2.2	38.1 ± 21.0	**3.8 ± 11.1**	− 3.36 ± 6.9	− 0.9 ± 4.1
C10	23.7 ± 12.3	− 0.2 ± 1.7	− 0.3 ± 1.6	− 0.1 ± 0.9	39.7 ± 28.0	0.9 ± 7.4	− 2.06 ± 5.9	− 1.4 ± 2.9
D1	29.7 ± 13.4	0.3 ± 1.8	0.5 ± 2.1	0.2 ± 1.3	26.0 ± 19.8	0.3 ± 7.1	− 3.36 ± 5.9	− 1.1 ± 3.9
D2	36.1 ± 12.8	− 0.3 ± 2.5	0.2 ± 3.9	− 0.2 ± 1.5	40.8 ± 24.8	0.3 ± 7.4	− 3.26 ± 6.1	− 1.3 ± 3.8
D3	21.9 ± 10.4	− 0.2 ± 1.6	0.7 ± 2.5	0.0 ± 1.0	39.3 ± 38.2	− 1.2 ± 08	− 2.96 ± 11.3	− 2.0 ± 4.4
C9-Scout view	**26.0 ± 9.2**	− **0.7 ± 4.08**			**38.1 ± 21.0**	**0.01 ± 14.97**		
C8-Scout view	**21.7 ± 10.2**	− **1.0 ± 3.20**			**38.1 ± 21.0**	**0.0 ± 9.40**		
C4-Scout view	**29.2 ± 11.6**	**0.3 ± 5.80**			**34.2 ± 22.3**	**0.1 ± 10.60**		

The bolded cells reflect the results of delimitation by means of AP projections for the same centers where AP scout-views

The negative values indicate missing the lung, whereas the positive values indicate overscanning. The last row is for the results of fine-tuning the network on AP scout-view images for center C9

**Fig. 5:**
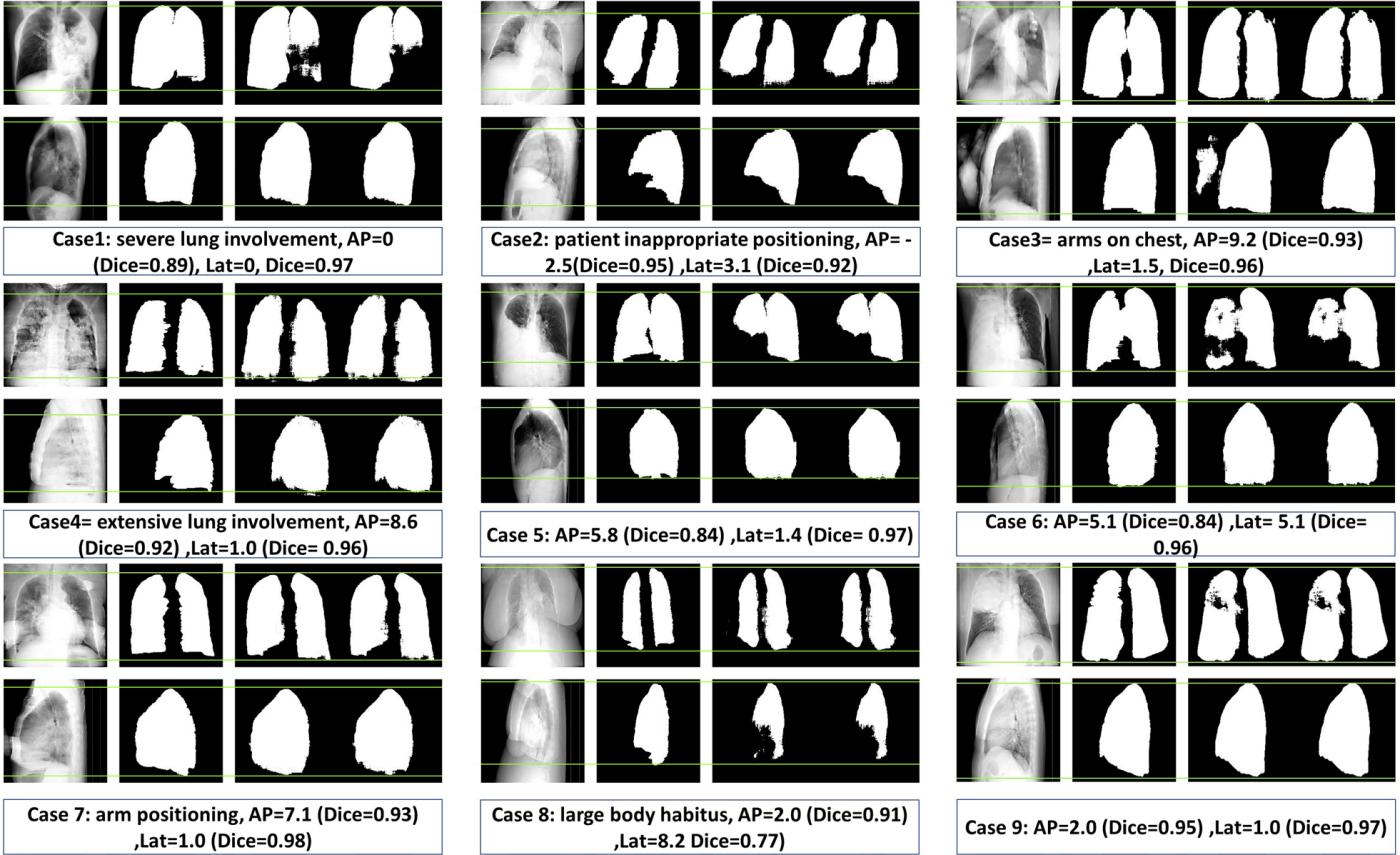
Examples of segmentation by the DL model in challenging cases. From the left to right: the projection image, the ground truth segmentation, the output of the DL network segmentation, the segmentation after post-processing. The upper row is for AP, whereas the lower row is for the lateral view. The error (Dice) for AP and lateral views are reported in the bottom of each panel. The line (green) shows the desired exact lung boundaries in the inferior and superior directions

Figure 5 illustrates some challenging cases with good agreement between DL and ground truth segmentation presenting with severe pathologic conditions, such as extensive pneumonia or collapsed lung(s), patients with inappropriate positioning, or overweight patients. As can be seen, the lung tissues were accurately distinguished. Additional file 1: Fig. 1 shows the performance of the deep neural network compared to human range selection for the outlier cases. Additional file 2: Fig. 2 shows sample images from the outlier group.

### Radiation dose estimates

Table 4 summarizes the selected average organ doses and ED resulting from three scan range selection scenarios. The ED and all out of lung field organ doses were significantly lower for the DL scan range compared to the human selected range (*p* < 0.001). The radiation dose was reduced for the thyroid, spleen, salivary glands, liver, adrenal, and oral mucosa by 58.2%, 60%, 94%, 47%, 45%, and 71%, respectively. Moreover, a 21% (1.3 mSv) dose reduction in terms of total body ED was achieved.

**Table 4:** Organ radiation doses (in mGy) and ED (in mSv) delivered to a patient for three different scenarios

Scenario		ED	CTDI_vol_	DLP	Adrenals	Breast	Esophagus	ETR	GB	Kidneys	Liver	LN	OM	Pancreas	RBM	SG	Skeleton	Skin	SI	Spleen	Stomach	Thymus	Thyroid
Exact Lung	mean	4.9	10.37	276	7.2	9.7	10.1	1.3	1.7	1.7	5.2	3.0	0.4	4.8	1.2	0.5	8.3	2.7	0.2	4.3	3.6	16.4	4.6
	STD	2.39	5.37	142	5.0	6.4	4.8	0.8	1.1	1.2	3.3	1.4	0.2	3.2	0.6	0.3	3.9	1.3	0.1	3.0	2.4	7.5	2.9
	Min	1.12	1.90	50	0.9	1.6	2.2	0.2	0.2	0.1	0.8	0.7	0.1	0.7	0.3	0.1	2.0	0.6	0.0	0.5	0.5	3.3	0.5
	Q1	2.9	5.93	154	3.2	4.6	6.5	0.6	0.8	0.6	2.5	1.9	0.2	2.2	0.7	0.3	5.1	1.5	0.1	1.9	1.6	10.6	1.9
	Q2	4.72	10.11	256	5.5	7.8	9.8	1.2	1.4	1.3	4.2	2.9	0.3	3.9	1.1	0.4	8.0	2.4	0.2	3.5	3.0	16.4	4.4
	Q3	6.34	13.61	365	10.4	14.5	12.8	2.0	2.4	2.5	7.2	3.9	0.5	6.8	1.5	0.6	10.8	3.5	0.3	6.2	5.1	20.8	6.9
	Max	12.47	22.10	662	22.4	25.4	24.0	4.1	6.1	6.4	16.3	7.2	1.2	16.4	2.7	1.5	19.4	6.4	0.7	15.0	12.2	34.6	15.1
Human	Mean	5.93	10.31	336	10.5	9.8	11.5	2.3	4.1	4.6	8.3	3.7	0.7	8.1	1.4	0.9	9.7	3.3	0.6	7.7	6.6	16.6	7.8
	STD	3.07	5.37	182	6.1	6.6	5.5	1.7	3.7	4.4	5.1	1.9	0.5	5.2	0.7	0.7	4.8	1.8	1.0	5.2	4.7	7.8	6.0
	Min	0.4	0.67	22	0.4	0.0	1.0	0.1	0.1	0.1	0.4	0.3	0.0	0.3	0.1	0.0	0.9	0.3	0.0	0.2	0.2	1.4	0.3
	Q1	3.5	5.91	185	5.3	4.6	7.4	1.1	1.6	1.5	4.2	2.2	0.3	3.9	0.9	0.4	6.0	1.8	0.2	3.7	3.2	10.9	3.9
	Q2	5.7	10.07	312	10.3	7.9	11.3	2.0	3.0	3.3	7.6	3.6	0.5	7.2	1.3	0.7	9.4	3.1	0.4	6.7	5.6	16.7	7.2
	Q3	7.74	13.57	448	14.3	14.7	14.6	3.3	5.5	6.4	11.3	4.8	0.9	11.3	1.8	1.2	12.7	4.4	0.7	10.9	9.2	21.2	10.4
	Max	14.82	21.60	825	25.6	25.6	25.9	9.7	20.8	22.5	22.7	8.9	4.3	22.1	3.1	5.1	22.0	8.2	7.8	22.0	22.1	35.0	34.1
DL	Mean	4.9	10.37	277	7.2	9.6	10.1	1.3	1.7	1.7	5.2	3.0	0.4	4.8	1.2	0.5	8.3	2.7	0.2	4.3	3.6	16.3	4.6
	STD	2.44	5.38	144	5.1	6.4	4.9	0.8	1.2	1.3	3.4	1.4	0.2	3.3	0.6	0.3	3.9	1.4	0.1	3.1	2.5	7.6	2.9
	Min	0.39	1.42	24	0.4	0.0	0.9	0.1	0.1	0.1	0.3	0.3	0.1	0.3	0.1	0.1	0.9	0.3	0.0	0.2	0.2	1.4	0.2
	Q1	2.95	6.00	155	3.2	4.6	6.5	0.6	0.8	0.6	2.5	1.9	0.2	2.2	0.8	0.3	5.2	1.5	0.1	1.9	1.6	10.7	1.9
	Q2	4.75	10.11	257	5.6	7.8	9.8	1.2	1.4	1.3	4.3	2.9	0.3	3.9	1.1	0.4	8.0	2.5	0.2	3.5	3.0	16.4	4.4
	Q3	6.36	13.62	367	10.5	14.5	12.9	2.0	2.4	2.5	7.3	3.9	0.5	6.9	1.5	0.6	10.8	3.5	0.3	6.3	5.2	20.8	7.0
	Max	12.47	22.10	662	22.4	25.4	24.0	4.1	6.1	6.4	16.3	7.2	1.2	16.4	2.7	1.5	19.4	6.4	0.7	15.0	12.2	34.6	15.1

*GB* Gall bladder, *RBM* Red Bone Marrow, *SI* Small Intestine, *SG* Salivary Glands, *ETR* Extra Thoracic Region, *LN* Lymph Nodes

**Fig. 6:**
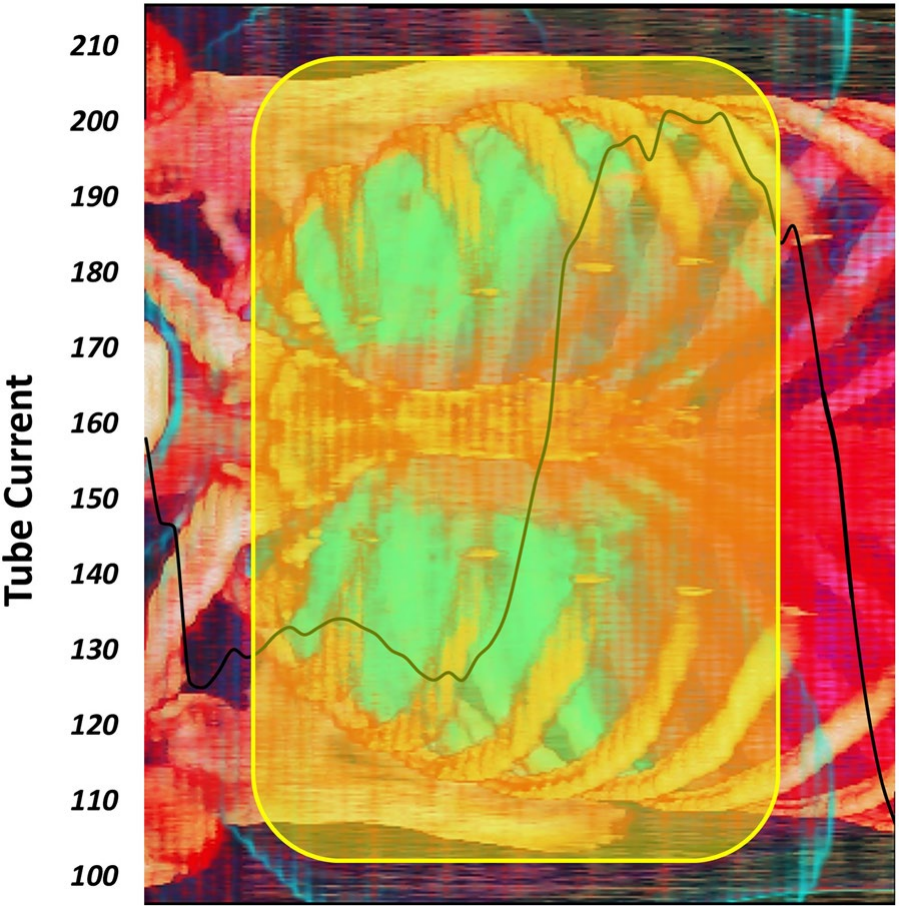
Tube current pattern (black line—mA) in the craniocaudal (left to right) direction. The yellow box indicates the lung segment boundaries

Figure 6 presents the tube current pattern for a female patient. The yellow highlighted region shows the lung region based on the 3D image. The tube current pattern (black line) shows increased tube current in the craniocaudal or Z-axis direction in body parts with more attenuation due to bony structures, larger diameter or both.

**Fig. 7:**
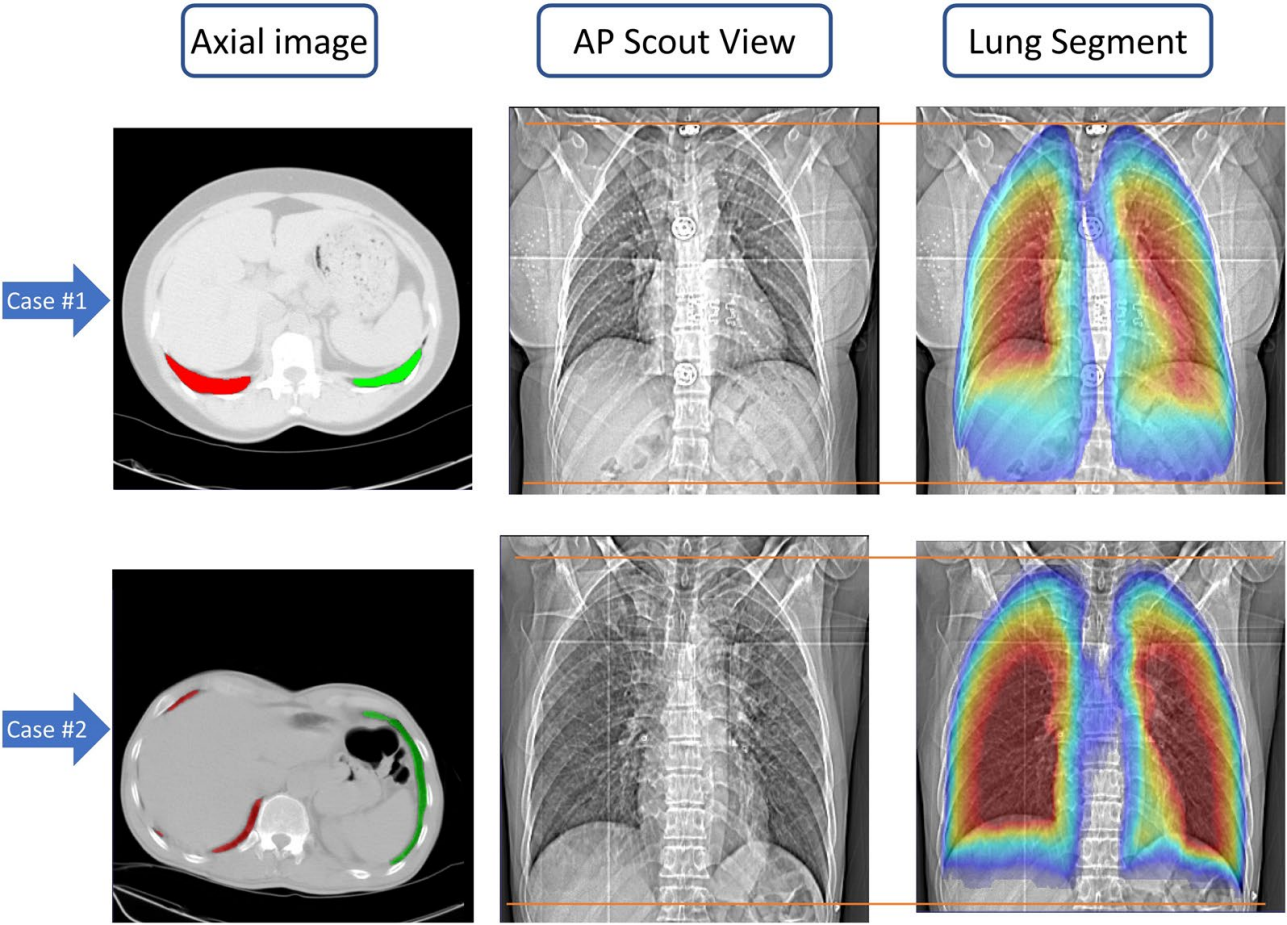
Display of the axial CT image and the segmented lungs (left), AP scout view (middle) and the segmented lung overlaid on the AP scout view (right) for two clinical studies. The red line indicates the desired scan limit in the inferior direction. The colored segments show the thickness of the lung segment in any region, where the red and blue colors stand for the thicker and narrower lung segments, respectively

Figure 7 presents the lung segments on AP scout view images and their position on the 3D image for two different patients. As can be seen, the lung segment is very narrow in the lung's inferior parts, while the condition can be different in the central parts far from costophrenic angles. The first study (upper row) shows a patient in which a narrow lung segment is extended to the inferior parts while in the second one (lower row), the lung segment is limited to the region shown on the AP scout view image. The colored segment shows the thickness of the lung, where the red color stands for a thicker and the blue color for a narrower lung segment. ("JET" color map lookup table).

## Discussion

Overscanning owing to issues linked to scan range delimitation has been noticed not only in chest CT but also in the abdomen and other body regions[34, 35]. Following the COVID-19 pandemic, the number of chest CT examinations has dramatically increased, which will have an undeniable impact on public medical exposure [36]. In this work, we investigated the incidence rate and extent of overscanning as well as its associated dosimetric impact. We evaluated a large-scale database composed of 20,820 patients from multiple centres and scanner models consisting of patients presenting with different pathologies to build a robust model for automatic scan range selection. A deep learning-assisted segmentation of the lungs was adopted to enable choosing the exact scan range to optimize patients' radiation dose associated with CT examinations [17]. We evaluated our methodology in terms of accuracy in range selection and radiation dose reduction. In addition, since the geometry and image quality of the scout-view images differ from CT spiral images, the generalizability of the proposed method in clinical setting was demonstrated by transferring and fine-tuning the trained network on AP projections to scout view images collected from one of the datasets.

Overscanning occurred in most cases (more than 95%), and the length of overscanning was more considerable in the inferior direction, which led to more contribution in patient dose, consistent with observations made in previous works [6, 7, 37]. The frequency of incidence and length of overscanning depends on the criteria based on which overscanning is defined. Demircioglu et al*.* [16] considered 10 mm tolerance range, while Cohen et al*.* [9] accepted overscanning less than 2 cm. Conversely, Yar et al*.* [6] defined the exact lung coverage as the ideal scan range. The vague anatomy of the lower margins and costophrenic angles, especially in patients with pathologies, overweight bodies, or additional devices on the body, such as respiratory aid devices, makes it more difficult, which may cause errors in distinguishing the exact border on the localizer image. Besides, the pressure on the technologists to avoid excluding parts of the lungs and missing any essential diagnostic information might force them to select a lower larger limit in the inferior direction.

Significant positive correlations with patients’ size and overscanning length were observed in scan ranges selected either by the operator (Human) or DL segmentation, i.e., the error was higher in patients with larger bodies (*p* < 0.001). Previous studies reported a similar correlation with the body mass index (BMI) or the thickness of subcutaneous fat [6, 9, 38].

The estimated organ doses revealed that some organs, such as the thyroid, liver, and gall bladder, which can be excluded in the chest scan range, receive unjustified and non-optimized radiation doses. In this study, we reported 67% (3.12 mSv) additional radiation dose to thyroid between Schwartz et al. [8] (0.35 mSv) and Zanca et al. [7] (5.1 mSv, 99%) studies. Overscanning was significantly more frequent in older patients in both directions. Yar et al*.* reported a positive and negative correlation with patient’s age for overscanning in inferior and superior directions, respectively [6]. Contrary to Cohen et al., our results seem to indicate that overscanning was more frequent in female patients [9].

The tube current pattern in the craniocaudal direction strongly depends on patient's body habitus and scanning parameters [39]. As presented in Fig. 6, due to the presence of bony structures in the superior direction and belly shape variations, the TCM system might affect the organ doses. Liao et al*.* reported that higher DLP values caused by overscanning are obtained when TCM is activated (20% vs. 56%) [38]. We used separately averaged tube currents for each range selection scenario to yield more realistic results. This critical issue was overlooked in previous studies.

One of the strengths of our adopted methodology is that the ground truth is accurately defined based on 3D axial CT slices, similar to the study conducted by Schwartz et al*.* [8], though our pipeline was fully automated. The advantage of this approach is that it does not miss any slice containing a small lung segment, which might happen on the localizer images, even for an experienced technologist. Figure 7 shows an example of lung shape in this region and the appearance on the localizer. The narrow lung segments in the most inferior parts are not recognizable owing to low contribution to photon attenuation and low contrast. In complicated cases, even an experienced radiologist might have doubts regarding the lower limit and accept overscanning to avoid missing anything that might cause misdiagnosis. This fact is overlooked in previous studies pertaining to automatic scan range selection using machine learning [14, 16, 40, 41].

The recent study by Demircioglu et al*.* reported on the use of a cGAN network for automatic scan range selection on the localizer image for chest CT images [16]. The desired scan range selected by the radiologist was used as ground truth to train the network. While the positive error (overscanning) obtained by the network compared with ground truth was considered correct range selection. In comparing the scan range chosen by the radiologist and the correct lung slice located on 3D axial images, there were 16.6 ± 17.3 mm (superior) and 22.7 ± 16.5 mm (inferior) differences between them. At the same time, they used the radiologist’s segment, which was prone to error, as ground truth for training the network and gained the average error equal to 1.8 ± 1.9 mm and 3.3 ± 5.6 mm for superior and inferior directions, respectively. An important conclusion of this work is that even two radiologists with 15 and 3 years of experience operating in rather calm conditions in the framework of a research project far from the pressure of clinical routine were not able to define accurately the scan range on the localizer image. This fact suggests the critical need for automated scan range detection algorithms.

The study of Huo et al*.* [40] employed a UNET network and reported − 1.68 ± 1.69% and 2.54 ± 2.31% error in superior and inferior directions, which can be equal to 4.2 ± 4.22 mm and 6.35 ± 5.75 mm in superior and inferior directions, respectively. Our proposed method outperformed available methods by yielding errors less than 2.5 mm and 1 mm in the inferior and superior directions, respectively.

The Dice and average range selection accuracy were much better when considering the lateral localizer. Using both AP and lateral localizers produce more reliable results as reported by Schwartz et al*.* [8], where they excluded patients with severe pathologies. We calculated the ED due to an AP localizer by the same margins covering the upper and lower boundaries of the thorax region. The EDs for routine clinical scenario was less than 0.05 mSv per view. It appears an acceptable practice to afford the radiation dose of an extra localizer by acquiring both AP and lateral localizers to prevent significant radiation dose to patients from overscanning. The main limitation of this study was the generation of the localizer from axial CT slices.

It was concluded that the generalizability of the model strongly depends on the vendor of the CT scanner. Since each manufacturer has specific filters and image processing on the scout-view images, the pixel value for scout-view images are not quantitative and standardized as reported previously [41, 42]. To this end, we propose to fine-tune our trained core model for each vendor. We performed the fine tuning on two vendors including Phillips and Siemens Healthcare. The trained network on Siemens Emotion scanner produced reliable results on other Siemens Somatom models.

However, by testing the trained model after fine-tuning, our results showed that the trained model could be transferred to real localizer images using transfer learning. As shown in Table 3, the pattern of errors is almost the same, while the standard deviation of errors is larger (14 vs 11 mm). Yet, the model is reproducible as proven by the sample size used for training (16,600 vs. 2595 cases). In addition, the perceived resolution of projection images in the cranio-caudal direction was limited by the slice thickness of spiral acquisition (1 to 8 mm), while the resolution of scout-view images was 1 or 2 mm. Another factor that makes the error more significant in the scout-view scan range delimitation is the difference in respiratory phases during the scout view and the spiral CT scan. This can be easily overcome by acquiring the scout-view in the same respiratory phase (routinely end-inspiration breath-hold) as the spiral. The developed methodology can be directly implemented on the main imaging console for different manufacturers to show the selected scan range for axial image acquisition, as a support tool for the technologist. The performance is vendor-dependent and the trained network could be an additional software linked to the acquisition software which provides only the superior and inferior desired Z value (pixel or slice location) to the technologist.

## Conclusion

Overscanning causes noticeable unnecessary radiation dose to patients undergoing chest CT examinations. This work proposed a deep learning-based scan range selection from the scout scan to eliminate common occurrence of overscanning in chest CT imaging by radiologists/operators. The proposed approach was developed and evaluated on a large variety of chest CT images presenting with different pathologies, acquired at diverse centers, on various scanners, using different acquisition parameters. The proposed automated deep learning-based scan range selection minimizes the noticeable extra radiation dose by excluding unjustified body parts from the CT scan range.

## Supplementary Information

**Additional file 1.** Presents examples of outlier cases.

## Data Availability

The code used in this work is available upon request.

## Abbreviations

AP: Anterior–posterior
cGAN: Conditional generative adversarial network
CNN: Convolutional neural network
CT: Computed tomography
CTDI_vol_: Volumetric CT Dose Index
D_eff_: Effective diameter
DL: Deep learning
DLP: Dose length product
ED: Effective dose
Lat: Lateral
SSDE: Size-specific dose estimate
TCM: Tube current modulation
