# Supplementary material for: Deep learning-based fully automated Z-axis coverage range definition from scout scans to eliminate overscanning in chest CT imaging
Source: Insights Imaging. 2021 Nov 6;12:162. doi: 10.1186/s13244-021-01105-3 (PMC8572075; doi:10.1186/s13244-021-01105-3)
Supplement: Supplementary file 1 — Additional file 1. Presents examples of outlier cases. [file 13244_2021_1105_MOESM1_ESM.docx]

**ELECTRONIC SUPPLEMENTARY MATERIAL**

**Description of outliers**

Absolute errors (less or more than zero) more than 10 mm achieved by the DL method on AP or lateral projections were considered as outliers. This occurred in 72 cases out of the 4220 (less than 2%), mainly in the inferior direction. Supplementary Figure 1 shows the performance of the deep neural network compared to human range selection for the outlier cases. All challenging cases had vague inferior boundary. Supplementary figure 2 shows sample images from the outlier group. The left middle image is a case presenting with the lowest Dice coefficient in the external group (AP and lateral Dice coefficients of 0.66 and 0.43, respectively). In this case, the high involvement of the lung and increased opacity made the task more difficult. This confirms excellent model performance in terms of scan range delimitation in more than 98% of the cases. All outliers were reviewed, and the leading causes reported were arm position, presence of air in the abdomen adjacent to the lungs, or a very narrow segment of the lung in the inferior and posterior position. In contrast, as shown in supplementary figures 1 and 2, the DL performance was better than technologists for most cases


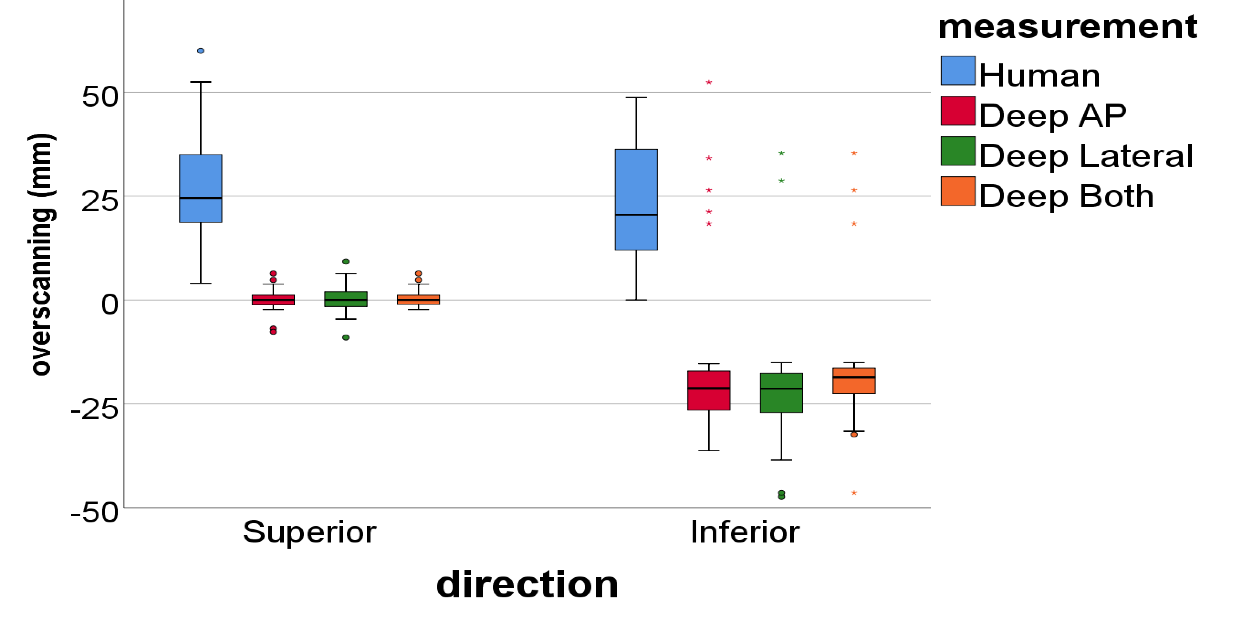


**Supplementary Figure 1.** The boxplot of error in superior (left) and inferior (right) directions in mm for 72 outlier cases.


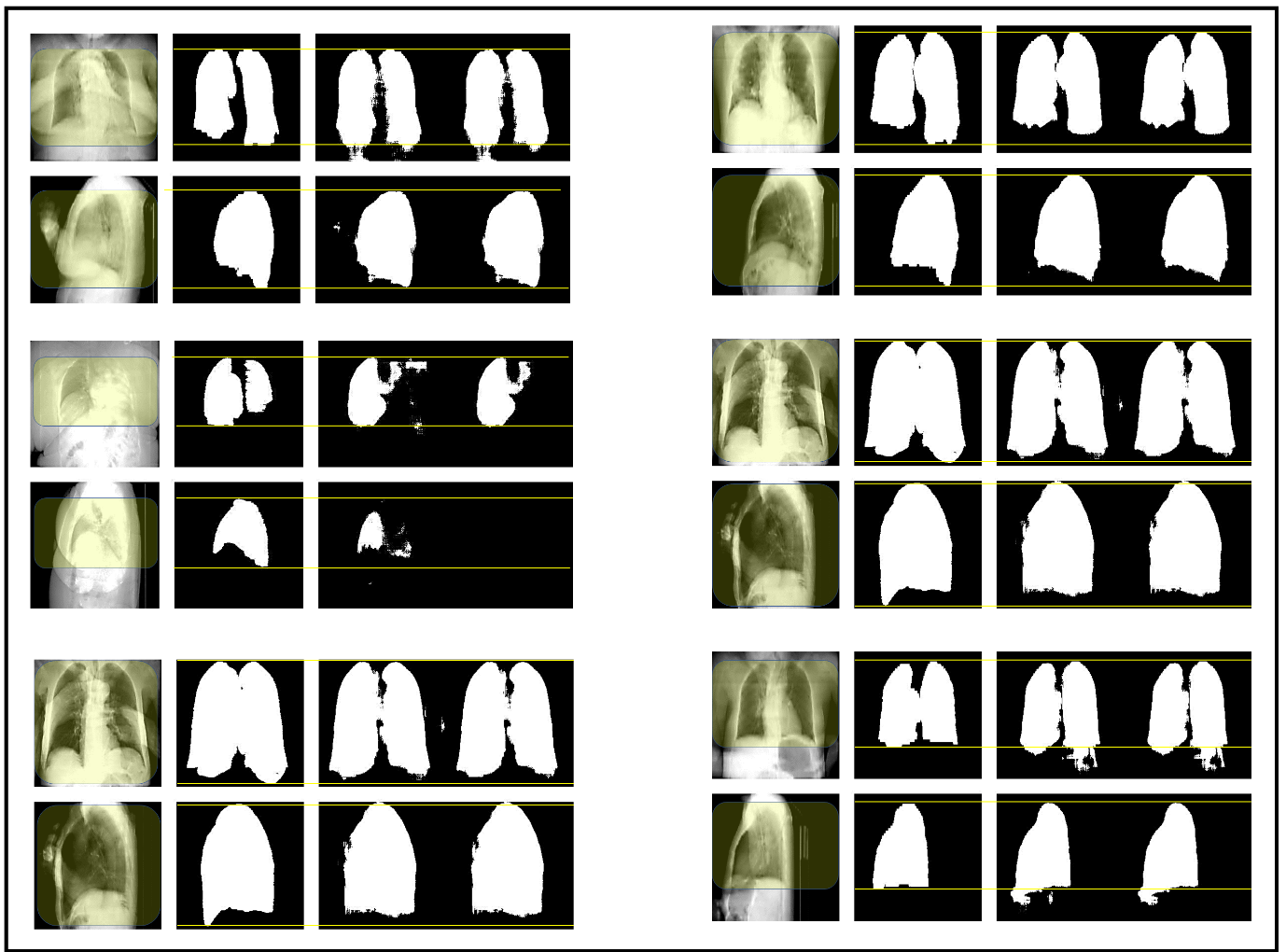


**Supplementary Figure 2.** Examples of outlier cases for AP (top) and lateral (bottom) projections. From left to right: the ground truth, DL, and post-processed segment. The line (yellow) and the box show the desired scan ranges.
